# Use of a Specialist Telephone Consultation Line for Long COVID in Primary Care in British Columbia: Retrospective Descriptive Quality Improvement Study

**DOI:** 10.2196/57021

**Published:** 2026-02-10

**Authors:** Saniya Kaushal, Jastinder Bhandal, Peter Birks, Jesse Greiner, Adeera Levin, Michelle Malbeuf, Zachary Schwartz

**Affiliations:** 1 Providence Health Care Research Institute and Provincial Health Services Authority, 1081 Burrard Street, Vancouver, BC, V6Z 1Y6, Canada, 1 6047511478; 2 Postgraduate Medical Education – Internal Medicine, Toronto Metropolitan University, Toronto, ON, Canada; 3 School of Medicine, University of Limerick, Limerick, Ireland; 4 Faculty of Medicine, The University of British Columbia, Vancouver, BC, Canada

**Keywords:** internal medicine, long COVID, COVID-19, SARS-CoV-2, GP, general practice, general practitioner, consult, respiratory, infectious, respiration, primary care, telephone, telehealth

## Abstract

**Background:**

Long COVID (post-COVID-19 condition) continues to challenge primary care. To support family physicians in British Columbia, the general internal medicine (GIM) COVID-19 Rapid Access to Consultative Expertise (RACE) line was launched in August 2020 to provide real-time specialist advice.

**Objective:**

This quality improvement study aimed to evaluate the implementation and utilization of the GIM-COVID-19 Long-Term Sequelae RACE line in British Columbia. Specifically, it sought to characterize the demographics of patients involved in RACE consultations, identify the most common themes and clinical queries presented by primary care providers, and assess how usage patterns evolved over time during the COVID-19 pandemic.

**Methods:**

We conducted a retrospective descriptive analysis of 149 RACE line call summaries between August 2020 and June 2021. Six calls were excluded due to insufficient information, such as incomplete documentation or absence of a clear COVID-19–related question. Because the original extraction notes are no longer available, further details about these calls cannot be provided, leaving 143 eligible calls. Data extracted included patient age, sex, geographical location, symptom type, and timing of symptom onset post–COVID-19 infection. Calls were categorized by symptom duration (acute: <2 wk, subacute: 2‐12 wk, chronic: >12 wk), thematic content (respiratory, fatigue, neurological, etc), and query type (symptom management, return-to-work, vaccination, etc). Data were coded independently by two reviewers using a standardized spreadsheet and predefined codebook. Discrepancies were resolved through discussion. Descriptive statistics summarized the findings.

**Results:**

Many calls involved female patients (91/143, 64%), with the most common age group being 40‐49 years (32/113, 28%). Most calls came from Greater Vancouver (35/83, 42%) and the Fraser Valley (29/83, 35%). Subacute symptoms (52/149, 35%) and vaccination-related concerns (29/149, 19%) were the most common inquiry types. Symptom-related inquiries accounted for 92 of 143 calls (64%), with 253 symptoms documented overall. Respiratory symptoms were most common (100/253, 40%), especially shortness of breath (35 calls), cough (26), and fatigue (23). Call volumes peaked from January to June 2021, coinciding with the provincial vaccine rollout.

**Conclusions:**

The GIM-COVID-19 Long-Term Sequelae RACE line served as a critical early support system for primary care providers as the long COVID landscape evolved. This quality improvement study emphasizes the value of rapid access and specialist-informed consultation tools during emerging public health challenges. The trends ascertained may inform future health system responses, particularly when designing more scalable, interdisciplinary models to support primary care in managing complex chronic conditions.

## Introduction

### Background

As COVID-19 transitions from a pandemic to endemic, its long-term effects, commonly referred to as long COVID, continue to place a substantial burden on both patients and health care systems. Real-time access to guidance for physicians has been offered through Rapid Access to Consultative Expertise (RACE) in British Columbia for over 12 years [1]. In August 2020, this service was expanded to include a long COVID RACE line, designed to support primary care physicians managing patients with persistent symptoms. Patients experiencing residual symptoms, such as shortness of breath, cough, and fatigue, for months postinfection are being diagnosed with postacute sequelae of SARS-CoV-2 (PASC) [2]. The World Health Organization defines post-COVID-19 condition as symptoms lasting at least two months and occurring usually three months from the onset of COVID-19, which cannot be explained by an alternative diagnosis [3]. In this study, data regarding patients with PASC collected through the general internal medicine (GIM)–COVID-19 Long-Term Sequelae division of the provincial RACE line were tabulated to identify trends in the long-term progression of COVID-19 symptoms. Findings of this study will offer information to improve the provincial patient care and long-term support for future patients with COVID-19, while offering insights on the usage of the GIM-COVID-19 Long-Term Sequelae RACE line.

As of June 27, 2022, the COVID-19 pandemic had affected over 544 million people worldwide [4]. The virus responsible for COVID-19 is SARS-CoV-2 [2]. Between its detection in December 2019 and June 27, 2022, the COVID-19 virus had infected 3.94 million Canadians [4]. British Columbia accounted for 373,974 of these cases [5]. Although over 369,000 British Columbians recovered from their acute COVID-19 illness, many are still experiencing residual symptoms for months or longer [5]. These patients have been deemed to have PASC [6]. This syndrome has many other names too, including long COVID, with some patients calling themselves long haulers; it is heterogeneous in presentation [2,6]. A 2022 *BMJ Open* meta-analysis approximated global PASC occurrence at 54% in hospitalized individuals and 34% in nonhospitalized patients, with an overall pooled occurrence of 43% [7]. In one study, nearly one-third of people who recovered from COVID-19 said they were still dealing with lingering symptoms that affected their day-to-day quality of life. Furthermore, recent US data suggests that about 1 in 7 adults have experienced symptoms of long COVID [8]. Common symptoms of long COVID include fatigue, dyspnea, chest pain, palpitations, cognitive dysfunction (“brain fog”), and anxiety, which may fluctuate or persist for months after acute infection [9]. Long COVID often flies under the radar in primary care. For example, in a large group of patients in England who had confirmed COVID-19, only about 1.8% were officially recorded as having long COVID, suggesting that many cases might be going unrecognized in everyday practice [10].

The underlying pathophysiology of long COVID is still being studied. However, contributions from immune dysregulation, viral persistence, microvascular dysfunction, and autonomic nervous system imbalance have been noted in several studies [11]. As of 2024, the National Institutes of Health RECOVER Initiative in the United States is conducting 8 clinical trials evaluating 13 potential interventions across 5 key symptom areas, with studies launched at over 50 sites nationwide to investigate treatments for long COVID [12,13]. Despite the increasing research, primary care providers often lack confidence in managing long COVID. A 2023 survey of 53 general practitioners (GPs) in Ireland found that only 8% felt confident in diagnosing long Covid and 81% were not confident in managing it, with 70% unaware of referral indications and 93% reporting educational gaps [14]. These findings highlight the pressing need for well-defined referral pathways and timely specialist involvement to support GPs in managing this complex condition effectively.

In British Columbia, Canada, the Post-COVID-19 Interdisciplinary Clinical Care Network (PC-ICCN) was developed to support the best outcomes for patients recovering from symptoms following COVID-19 infection through research, education, and clinical care [15]. One of the clinical resources within the PC-ICCN included the establishment of the GIM-COVID-19 Long-Term Sequelae division of the provincial RACE line [15]. This resource provides immediate (<2 h) specialist advice to GPs caring for patients with long-term sequelae of COVID-19 infection. The provincial RACE line has existed in British Columbia since 2010 and provides access to immediate specialist advice/consultation across the province [1].

The GIM-COVID-19 Long-Term Sequelae RACE line is answered by a dedicated group of GIM specialists with an interest in and experience with acute and chronic COVID-19 [1]. The guidance provided by the GIM physicians includes diagnostic investigations, management, and navigation of these complex patients. Since its inception, the RACE line has aimed to bridge gaps in access to timely specialist input as an approach intended to reduce unnecessary referrals and support primary care providers in managing complex conditions [16]. In Ontario, a special eConsult service for long COVID helped family doctors get quick advice from specialists, which meant patients got the help they needed faster, often without having to see a specialist in person [17].

Similar post-COVID advice or consultation pathways have been established in countries such as the United Kingdom (NHS long COVID clinics), the United States (National Institutes of Health RECOVER Initiative), and Australia (long COVID management guidelines for GPs), reflecting global recognition of the condition’s complexity and the need for specialist support [10,18,19].

This report presents an analysis of the types and frequencies of calls made to the GIM-COVID-19 Long-Term Sequelae RACE line. This analysis enables the identification of trends in patient presentations, primary care practitioner concerns, and related questions. This data informs on the development of education, tools, and care plans, which improves the quality of care and long-term support for patients with COVID-19 and their health care providers.

### Objectives

The aim of this study was to evaluate the usage patterns, themes of inquiry, and demographic data associated with the GIM-COVID-19 Long-Term Sequelae RACE line in British Columbia. By analyzing call content and frequency, we sought to identify knowledge gaps among primary care providers and inform future improvements in post–COVID-19 care resources and communication strategies.

## Methods

### Overview

This is a quality improvement study evaluating the GIM-COVID-19 Long-Term Sequelae RACE line data from its launch in August 2020 to June 2021. These data are comprised of the documented exchanges between primary care practitioners (PCPs) and GIM specialists. The study received quality improvement approval through Providence Health Care and was exempt from formal research ethics board review. No patient-identifying data were accessed, and all analysis was conducted on anonymized call notes.

### Data Source and Call Selection

In total, 149 RACE line call medical notes were systematically reviewed to extract data regarding the variables of interest: patient demographics (age, sex, region) and types of queries related to COVID-19 (acute symptoms, subacute symptoms, chronic symptoms, vaccination inquiries, miscellaneous questions). The data from these calls were tabulated for analysis. Call notes were reviewed manually using a standardized spreadsheet for data extraction. Each variable was assigned a predefined codebook category, and disagreements were resolved through discussion and consensus. Six calls were excluded from this study because they were too vague to draw conclusive findings. Reasons for exclusion included incomplete documentation, lack of a clear COVID-19–related question, or insufficient clinical information to categorize the inquiry. Excluded calls were logged and reviewed to ensure consistent application of exclusion criteria. The remaining 143 calls were used to observe trends in age, sex, geographical location, types of queries, timing, and symptoms of patients of the GIM-COVID-19 Long-Term Sequelae RACE line between August 2020 and June 2021. All calls categorized under the GIM-COVID-19 Long-Term Sequelae RACE line service during this period were included; calls unrelated to post-COVID symptoms were excluded.

### Query Classification

For RACE calls regarding patient symptoms, we examined the reported symptoms according to the time post–COVID-19 infection. This was predetermined as 0‐2 weeks following diagnosis to represent acute COVID-19 symptoms, 2‐12 weeks following diagnosis to represent subacute COVID-19 symptoms, and >12 weeks following COVID-19 diagnosis to represent chronic COVID-19 symptoms. The relative frequencies of types of symptoms reported to the GIM-COVID-19 Long-Term Sequelae RACE line were analyzed and compared.

### Temporal Analysis of Calls

We also analyzed the reasons for calls to the RACE line by time period within the pandemic. First, we described the type of calls received during the different COVID-19 “waves” as occurred in British Columbia, including August to December 2020, January 2021 to March 2021, and April 2021 to June 2021. Second, we assessed the reasons for the call before and after the availability of COVID-19 vaccines. We hypothesized that the reasons for calls would vary depending on the time period during the pandemic. In addition, calls were ranked by the phase of the pandemic to explore trends in the volume and nature of queries, including vaccine-related concerns and the timing of chronic symptom presentations. Descriptive statistics (frequencies and proportions) were used to summarize query types by time period. The data were analyzed using Microsoft Excel (Microsoft Corp), which was then used to organize, summarize, and identify patterns in query types, symptom categories, and temporal trends.

### Symptom Categorization and Coding

Each call was examined to determine whether it included symptom-related content, which was then grouped by organ system (eg, respiratory, neurological, or gastrointestinal). In cases where more than one symptom was mentioned, all relevant details were recorded to capture the full scope of the patient’s concerns. When symptoms overlapped or were unclear, the team discussed them collectively before assigning categories to maintain consistency across the dataset.

Calls were coded based on symptom duration, query type, and organ system using a predefined framework (Table 1). The initial framework was adapted from published sources and refined after the pilot coding of 10 calls. Two reviewers independently applied this framework to the complete dataset, meeting regularly to compare interpretations and update the categories as new themes appeared. Any differences in coding were resolved through discussion until consensus was reached.

**Table 1.** Coding framework used to classify symptom duration, query type, and symptom system for RACE line calls.

Variable	Categories	Description/examples
Symptom duration	Acute (<2 wk), subacute (2‐12 wk), chronic (>12 wk)	Based on time since acute COVID-19 infection
Query type	Symptom management, vaccination, return-to-work/school, diagnosis clarification, medication advice, other	Categorized according to the primary purpose of the call
Symptom system	Respiratory, fatigue, neurological, cardiovascular, mental health, gastrointestinal, multisystem, other	Grouped by system affected, based on physician documentation

Although formal interrater reliability statistics (such as κ values) were not calculated, the team held multiple calibration meetings to ensure categories were applied consistently. All coded data were entered into a structured Microsoft Excel sheet for organization and analysis. Quantitative findings were summarized descriptively, while qualitative insights were drawn inductively from the free-text notes accompanying each call.

### Thematic Analysis

A qualitative content analysis was carried out to explore recurring themes in the clinical questions and patient symptom narratives. Rather than relying on a preset framework, themes were drawn directly and inductively from the anonymized call notes. This approach helped the research team capture how primary care providers’ clinical concerns and informational needs evolved over time while managing patients with post–COVID-19 conditions during the study period.

### Ethical Considerations

This project was conducted as a quality improvement initiative and did not involve direct interaction with patients or the collection of personal patient information that would allow identification. Thus, it did not require formal review by a research ethics board. This project was reviewed and approved as a quality improvement initiative by Providence Health Care and was deemed exempt from formal institutional research ethics board review.

All the data analyzed were anonymized call summaries obtained from the RACE line, which documents virtual consultations between PCPs and GIM specialists. No identifiable or sensitive personal health information was accessed, and there were no links to patient charts or follow-up data.

Individual informed consent was not required since this was a secondary analysis of anonymized and nonidentifiable data. No participants were directly involved or contacted for the purposes of this study. The original data source did not include participant-level identifiers or contact information. No compensation was offered to anyone, as this study did not include human subjects or involve any form of participant recruitment. No images or materials in this manuscript include identifiable individuals.

## Results

### Patient Demographics

Table 2 outlines the characteristics of patients whose primary care providers contacted the RACE line between August 2020 and June 2021. The majority of calls concerned patients in middle age, most commonly those between 40 and 49 years (28%), followed by individuals aged 50‐59 years (23%) and 30‐39 years (16%). Women represented nearly two-thirds of all patients (64%), while men accounted for just over one-third (36%).

Geographically, calls were concentrated in the more urban and suburban regions of British Columbia, particularly Greater Vancouver (35/83, 42%) and the Fraser Valley (29/83, 35%)—together making up more than three-quarters of all consultations. Smaller proportions of calls came from Vancouver Island (8/83, 10%), with the remainder originating from rural and remote regions, including Northern British Columbia (4/83, 5%) and the Interior (3/83, 4%), as well as from out of province (Yukon: 3/83, 4%; Alberta: 1/83, 1%).

**Table 2.** Demographics of COVID-GIM-Post-Infection Care RACE line patients. Percentages may not total 100 due to rounding.

Variable	Category	Patients, n (%)
Age group, years (n=143)		
	<20	3 (2)
	20‐29	8 (7)
	30‐39	18 (16)
	40‐49	32 (28)
	50‐59	26 (23)
	60‐69	9 (7)
	70‐79	13 (12)
	≥80	5 (4)
Sex (n=143)		
	Male	52 (36)
	Female	91 (64)
Geographic region (n=83)		
	Greater Vancouver	35 (42)
	Fraser Valley	29 (35)
	Vancouver Island	8 (10)
	Northern British Columbia	4 (5)
	Interior British Columbia	3 (4)
	Yukon	3 (4)
	Alberta	1 (1)

### Types of Queries Received

**Figure 1.**
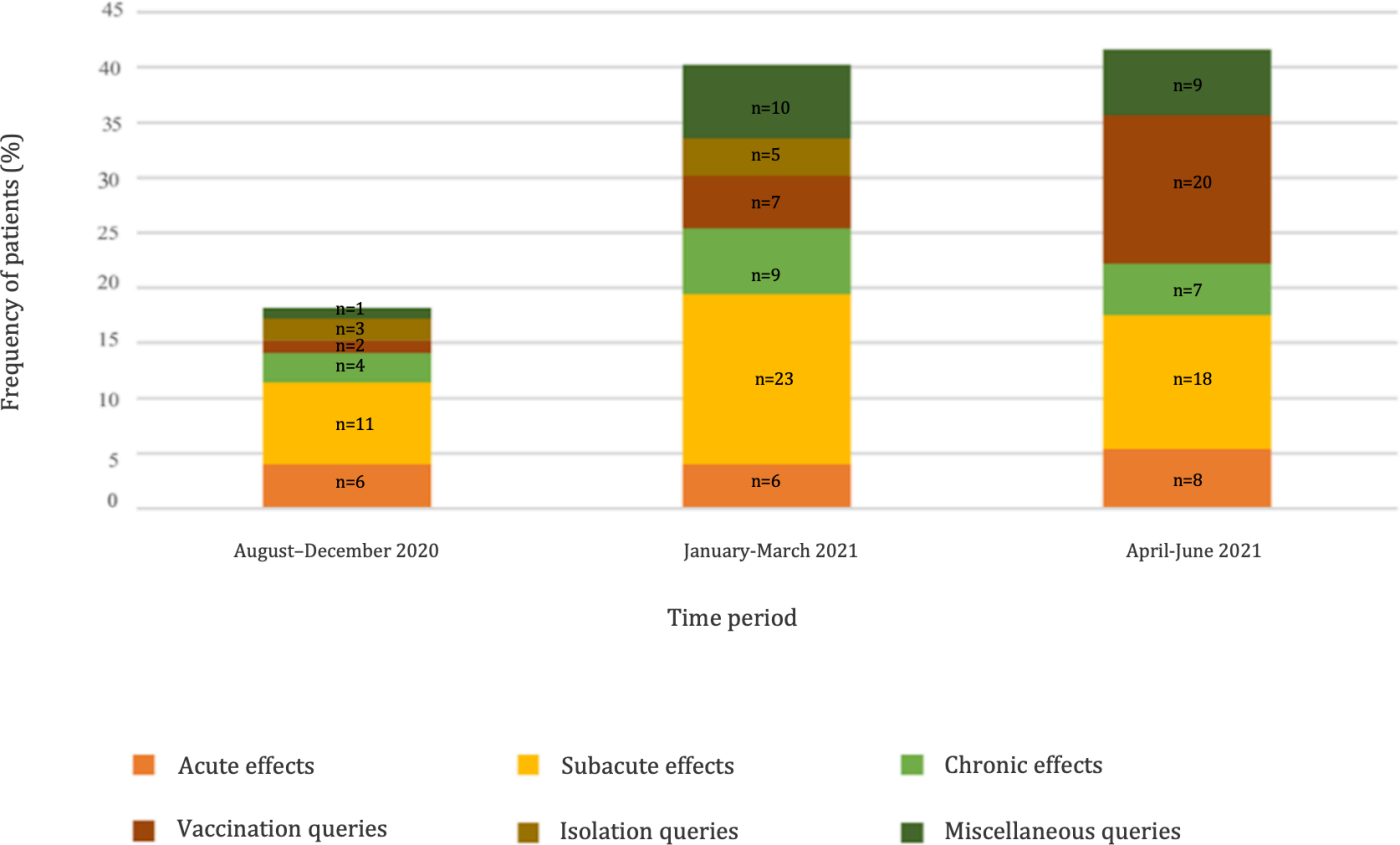
Progression of types of COVID-19–related queries to the COVID-GIM-Post-Infection Care RACE line by time period. RACE: Rapid Access to Consultative Expertise.

The types of COVID-19–related queries that were received by the COVID-GIM-Post-Infection Care RACE line are summarized by time period. The data demonstrate that subacute (2‐12 wk following diagnosis) symptoms were cited in 52 of 149 calls (35%) and vaccination queries were cited in 29 calls (19%), making both the most common RACE line call matters. A visual breakdown of these query types by time period is provided in Figure 1.

### Timing of RACE Line Queries

As shown in Figure 1, there was a much larger frequency of calls from January to March 2021 (n=60, 40%) and April to June 2021 (n=62, 42%) compared to the earlier 27 (18%) calls from August to December 2020. There was a much larger number of queries following the introduction of the COVID-19 vaccine (n=122, 82%) compared to the time period prior (n=27, 18%). This increase in queries temporally coincided with the start of the vaccine rollout in early 2021.

### Symptoms Reported by System and Time Frame

Symptoms reported to the GIM-COVID-19 Long-Term Sequelae RACE line between August 2020 and June 2021 were compiled. Data from the calls that reported symptoms present 2‐12 weeks following COVID-19 diagnosis (subacute) or >12 weeks following COVID-19 diagnosis (chronic) are summarized visually in Table 3.

**Table 3.** Symptoms reported to the COVID-GIM-Post-Infection Care Rapid Access to Consultative Expertise line by system and category.

System/category	Values, n (%)
Subacute symptoms (2‐12 wk following diagnosis)	
Respiratory	71 (28.1)
Malaise/fatigue	35 (13.8)
Neurological	17 (6.9)
Cardiac	16 (6.4)
Musculoskeletal	10 (3.9)
Gastrointestinal	6 (2.5)
Neuropsychological	6 (2.5)
Abnormal laboratory/diagnostic findings	0 (0)
Genitourinary	4 (1.5)
Autoimmune	0 (0)
Dermatological	0 (0)
Chronic symptoms (≥12 wk following COVID-19 diagnosis)	
Respiratory	29 (11.3)
Malaise/fatigue	11 (4.4)
Neurological	9 (3.4)
Cardiac	9 (3.4)
Musculoskeletal	9 (3.4)
Gastrointestinal	5 (2)
Neuropsychological	4 (1.5)
Abnormal laboratory/diagnostic findings	5 (2)
Genitourinary	0 (0)
Autoimmune	3 (1)
Dermatological	3 (1)
Combined frequency of reported symptoms following COVID-19 infection (n=253)	
Respiratory	100 (39.5)
Malaise/fatigue	46 (18.2)
Neurological	26 (10.3)
Cardiac	25 (9.9)
Musculoskeletal	19 (7.5)
Gastrointestinal	11 (4.3)
Neuropsychological	10 (4)
Abnormal laboratory/diagnostic findings	5 (2)
Genitourinary	4 (1.6)
Autoimmune	3 (1.2)
Dermatological	3 (1.2)

Questions about specific symptoms varied by time postinfection, but the most frequent symptoms across time periods included shortness of breath, cough, and fatigue. Overall, 92 (62%) calls focused on symptoms; within these calls, there were 253 total symptoms reported. Shortness of breath was the most reported symptom, being identified in 35 calls (38%). Cough, fatigue, and fever were the other highest reported symptoms recorded among GIM-COVID-19 Long-Term Sequelae RACE line patients, accounting for 26 calls (28%), 23 calls (25%), and 22 calls (24%), respectively. The most common symptoms reported 12 weeks postinfection were shortness of breath, chest pain, and fatigue.

Table 3 organizes the symptoms by body system, allowing for recognition of symptom clusters and trends. Our findings demonstrated that respiratory, malaise/fatigue, and neurological symptoms were the most common categories of postinfection symptoms resulting in calls to the RACE line. Respiratory symptoms made up 40% (100/253) of reported symptoms. Respiratory symptoms reported included cold, coryza, cough, hypoxemia, lung infiltrate, nasal congestion, rhinorrhea, shortness of breath, sore throat, sputum, and wheezing. Shortness of breath, cough, and hypoxemia were the most persistent respiratory symptoms listed. Neurological symptoms described by PCPs regarding their patients included dizziness, light-headedness, numbness, vertigo, and paresthesia. The wide range of symptoms reported reinforces the multisystemic nature of long COVID syndromes and the complexity of clinical management in primary care settings.

## Discussion

### RACE Line Utilization and Demographics

This report provides information on the use of the GIM-COVID-19 Long-Term Sequelae RACE line, specifically between August 2020 and June 2021. Our data suggest that the RACE line has been a utilized resource for PCPs, especially in the Greater Vancouver and Fraser Health regions. The data obtained indicate that the frequency of calls to the RACE line has increased throughout the pandemic. Although this RACE line is accessible across the province, there was minimal uptake outside the two aforementioned regions, as seen in Table 2. This may reflect “burden” and populations affected or an underutilization by more sparsely populated regions. Further analysis of unmet need versus not needed is required so that we can ascertain if different strategies for awareness of the RACE line outside of highly populated areas is required. This study highlights the need to understand mechanisms by which GPs learn about new RACE lines and this resource in particular. These findings highlight the opportunity to use centralized consultation services like the RACE line more strategically during public health crises. As novel health conditions emerge, having specialist-access infrastructure already in place can ensure faster, provincewide dissemination of clinical support.

Observable trends in the data indicated that the most common age group of RACE line patients was 40‐49 years old, with 40 of 143 patients (28%) in this bracket. Females made up 91 of 143 patients (64%). Interestingly, these demographics are also representative of those mostly likely to be referred and seen in post-COVID recovery clinics across the province. Thus, information from clinicians to PCPs is based on relevant experience in a similar population. Future planning should include population-based targeting strategies and outreach initiatives tailored to groups underrepresented in utilization patterns, including rural and Indigenous communities.

### Themes in Queries

RACE line subject matter involved acute, subacute, and chronic effects experienced by the patients. Respiratory, malaise/fatigue, and neurological symptoms were the most common categories of postinfection symptoms reported to the RACE line. This data can be used to identify gaps in PCP knowledge in the diagnosis and management of persistent symptoms following COVID-19 infection and has informed the development of educational resources for health care practitioners. Adopting these care plans would significantly improve the quality and aid provided by future RACE line calls in this division. Therefore, increasing the accessibility of resources on managing these identified symptoms should be a focus moving forward. This is an initiative that is currently being implemented by the provincial post-COVID recovery clinic websites. Other topics of interest include that RACE line calls also frequently centered around vaccinations, isolation periods, the impact of preexisting health conditions on COVID-19 manifestation, and antiviral treatments. This is despite regular bulletins and updates to medical doctors from provincial health bodies, infectious disease specialists, and public health officers. This highlights key areas of uncertainty and opportunities for clearer messaging to health care practitioners. Our findings align with global surveys of GPs, such as those referenced in the Introduction, that identified a lack of confidence in managing long COVID, particularly around referral pathways and symptom assessment. The RACE line model may serve as a blueprint to help bridge these knowledge gaps and offer just-in-time support when new syndromes with unclear management approaches arise.

Our findings are consistent with trends observed internationally. In British Columbia, the RACE line was most often used for queries related to respiratory, fatigue, and neurological symptoms, as well as questions about vaccination and isolation. These areas of uncertainty are not unique to the province. Similar issues have been described elsewhere, prompting the creation of virtual and rapid-access models for post–COVID-19 care.

In the United Kingdom, Leeds launched one of the earliest integrated long COVID programs that combined a specialist multidisciplinary team, community rehabilitation services, and self-management resources, reflecting NHS England’s broader plan to expand post-COVID assessment clinics [20]. By late 2021, the NHS had established nearly 90 dedicated long COVID clinics across England, providing comprehensive multidisciplinary assessment and rehabilitation [21].

In the United States, the Johns Hopkins Post-Acute COVID-19 Team used telemedicine to coordinate care across multiple specialties without relying on a centralized physical clinic. This approach highlighted how virtual models can effectively manage complex cases and maintain accessibility [22].

In Australia, a Melbourne-based telehealth long COVID service has supported more than 500 patients nationwide, including those in rural and pediatric populations. Its collaboration with local primary care providers helped ensure equitable access despite geographical barriers [23].

Across Europe, similar models have been implemented. A national survey of 124 post-COVID clinics in Italy found that 93.5% maintained direct communication pathways with GPs, and nearly one-quarter incorporated telemedicine into their standard care processes [24].

Together, these initiatives reflect a shared global recognition of the need for coordinated, multidisciplinary approaches to long COVID care. Although the RACE line differs as a physician-to-physician consultation model, it aligns with these international strategies by promoting timely access to specialist input and integration within primary care networks.

### Study Strengths and Limitations

This study examines routinely standardized and collected information generated from RACE calls over an 11-month period of time, where a small number of dedicated individuals were answering calls. To our knowledge, this is the only provincial post-COVID RACE line set up in Canada. Of the 149 RACE line calls made to the GIM-COVID-19 Long-Term Sequelae RACE line during the time period being investigated**,** only 6 of 149 calls were excluded due to unclear documentation. Therefore, 143 calls (96% of all applicable calls) were used to generate the findings of this quality improvement study. This corresponds to a study that provides a greater overview of the GIM-COVID-19 Long-Term Sequelae RACE line calls than one with a lower percentage of population representation. This is indicative of sampling validity, and it translates to a greater elimination of design or inclusion bias. Prejudice in this study was also greatly eliminated as tabulation was performed by an outside party with no preexisting relationships with the subjects of the studied RACE line calls.

This study had some limitations. First, the preexisting medical conditions of PCPs’ patients were not taken into consideration when tabulating reported postinfection symptoms. Preexisting conditions may have exacerbated the prevalence of some symptoms in patients with long COVID-19. Second, there is no clear differentiation in the severity of symptoms described by PCPs regarding their patients and no standardized approach to measure these symptoms on RACE line calls. It is also not known if other specialist RACE lines were called for individuals with more specific organ system symptoms (eg, respirology, cardiology, psychiatry). Therefore, this study’s data may reflect a smaller number of true RACE calls for long COVID. Third, the retrospective nature of the study limits our ability to verify or clarify PCP interpretations of patient symptoms. Additionally, given the absence of severity scoring or follow-up outcomes, we cannot correlate RACE call content with long-term patient trajectories. Nonetheless, our use of deidentified service data and a near-complete inclusion rate minimizes bias and supports the robustness of thematic trends observed.

### Conclusion

The GIM-COVID-19 Long-Term Sequelae RACE line was introduced to provide PCPs with prompt medical advice from GIM specialists regarding chronic COVID. Over the course of this pandemic, this resource has been used by health care professionals to improve timely access to care, and it has provided support for the appropriate delivery of high-quality care by PCPs. This study investigated GIM-COVID-19 Long-Term Sequelae RACE line calls between August 2020 and June 2021, revealing many trends in the data. These calls mainly involved consults regarding the long COVID-19 symptoms being experienced by PCPs’ patients. Respiratory symptoms were the leading type of symptom reported, with shortness of breath, cough, fatigue, and fever being the most common, respectively.

Moving forward, RACE calls can be monitored in situations of emerging diseases to better inform and educate community physicians about common complaints that patients are presenting with. The infrastructure and success of the RACE line display the value of creating specialty-informed, swift, and scalable support services during potential future public health emergencies. Integrating rapid-access consultation lines within more broad interdisciplinary networks, such as the PC-ICCN, can enable the timely translation of knowledge and reduce primary care uncertainty for atypical or complex conditions. The burden of chronic and multisystemic conditions is increasing, even outside the pandemic, and future iterations of RACE models could be modified to support conditions like myalgic encephalomyelitis/chronic fatigue syndrome, postviral syndromes, or multimorbidity in aging populations. Policymakers and health system leaders should consider sustained funding and integration of virtual consult models as part of long-term primary care innovation. The experience in British Columbia reflects a pattern seen internationally. Health systems across the United Kingdom, the United States, Australia, and several European countries have developed comparable virtual or rapid-access pathways to manage post-COVID. Placing the RACE line within this wider global effort highlights its importance not only as a local quality improvement initiative but also as a practical example of how specialist consultation models can support the evolving response to complex long COVID care.

## Abbreviations

GIM: general internal medicine
GP: general practitioner
PASC: postacute sequelae of SARS-CoV-2
PC-ICCN: Post-COVID-19 Interdisciplinary Clinical Care Network
PCP: primary care practitioner
RACE: Rapid Access to Consultative Expertise

## References

[1] (2022). Rapid access to consultative expertise: an innovative model of shared care. RACE.

[2] (2022). Naming the coronavirus disease (COVID-19) and the virus that causes it. World Health Organization.

[3] (2021). A clinical case definition of post COVID-19 condition by a Delphi consensus, 6 October 2021. World Health Organization.

[4] Ritchie H, Mathieu E, Rodés-Guirao L, Appel C, Giattino C, Ortiz-Ospina E (2022). Coronavirus pandemic (COVID-19). Our World in Data.

[5] (2022). BC COVID-19 data. BC Centre for Disease Control.

[6] Proal AD, VanElzakker MB (2021). Long COVID or post-acute sequelae of COVID-19 (PASC): an overview of biological factors that may contribute to persistent symptoms. Front Microbiol.

[7] (2023). Global prevalence of post-acute sequelae of COVID-19 (PASC) or long COVID: a meta-analysis and systematic review. BMJ Open.

[8] Resendez S, Brown SH, Ruiz Ayala HS (2024). Defining the subtypes of long COVID and risk factors for prolonged disease: population-based case-crossover study. JMIR Public Health Surveill.

[9] Long COVID signs and symptoms. Centers for Disease Control and Prevention.

[10] Meza-Torres B, Delanerolle G, Okusi C (2022). Differences in clinical presentation with long COVID after community and hospital infection and associations with all-cause mortality: English Sentinel Network Database Study. JMIR Public Health Surveill.

[11] Castanares-Zapatero D, Chalon P, Kohn L (2022). Pathophysiology and mechanism of long COVID: a comprehensive review. Ann Med.

[12] (2023). NIH launches long COVID clinical trials through RECOVER initiative, opening enrollment. National Institutes of Health.

[13] Reviewing RECOVER’s impact in 2024. RECOVER COVID initiative.

[14] Farrell A, O’Flynn J, Jennings A (2024). An investigation into general practitioners’ experience with long COVID. Ir J Med Sci.

[15] (2025). About the PC-ICCN. Provincial Health Services Authority.

[16] About RACE. South Island Division of Family Practice.

[17] Singh J, Quon M, Goulet D, Keely E, Liddy C (2025). The utilization of electronic consultations (eConsults) to address emerging questions related to long COVID-19 in Ontario, Canada: mixed methods analysis. JMIR Hum Factors.

[18] (2023). The NHS plan for improving long COVID services. NHS England.

[19] Allard N, Miller A, Morgan M, Chakraborty S (2022). Post-COVID-19 syndrome/condition or long COVID: persistent illness after acute SARS CoV-2 infection. Aust J Gen Pract.

[20] Parkin A, Davison J, Tarrant R (2021). A multidisciplinary NHS COVID-19 service to manage post-COVID-19 syndrome in the community. J Prim Care Community Health.

[21] Greenhalgh T, Darbyshire JL, Lee C, Ladds E, Ceolta-Smith J (2024). What is quality in long covid care? Lessons from a national quality improvement collaborative and multi-site ethnography. BMC Med.

[22] Brigham E, O’Toole J, Kim SY (2021). The Johns Hopkins Post-Acute COVID-19 Team (PACT): a multidisciplinary, collaborative, ambulatory framework supporting COVID-19 survivors. Am J Med.

[23] Whyler N, Atkins L, Hogg P (2024). Harnessing the benefits of telehealth in long COVID service provision. Public Health Rev.

[24] Floridia M, Grassi T, Giuliano M (2022). Characteristics of long-COVID care centers in Italy. A national survey of 124 clinical sites. Front Public Health.

